# Active recharge biphasic stimulation for the intraoperative monopolar review in deep brain stimulation

**DOI:** 10.3389/fnhum.2024.1349599

**Published:** 2024-02-28

**Authors:** David Mampre, Min Jae Kim, Tucker Oliver, Zachary Sorrentino, Vyshak Chandra, Carlton Christie, Rasheedat Zakare-Fagbamila, Justin D. Hilliard, Joshua K. Wong

**Affiliations:** ^1^Department of Neurosurgery, University of Florida, Gainesville, FL, United States; ^2^Department of Neurosurgery, University of Pennsylvania, Philadelphia, PA, United States; ^3^Department of Neurosurgery, Medical College of Georgia, Augusta, GA, United States; ^4^Department of Neurology, Fixel Institute for Neurological Diseases, University of Florida, Gainesville, FL, United States

**Keywords:** deep brain stimulation, active recharge, charge balancing, biphasic, passive recharge

## Abstract

**Introduction:**

Charge balancing is used in deep brain stimulation (DBS) to avoid net charge accumulation at the tissue-electrode interface that can result in neural damage. Charge balancing paradigms include passive recharge and active recharge. In passive recharge, each cathodic pulse is accompanied by a waiting period before the next stimulation, whereas active recharge uses energy to deliver symmetric anodic and cathodic stimulation pulses sequentially, producing a net zero charge. We sought to determine differences in stimulation induced side effect thresholds between active vs. passive recharge during the intraoperative monopolar review.

**Methods:**

Sixty-five consecutive patients undergoing DBS from 2021 to 2022 were retrospectively reviewed. Intraoperative monopolar review was performed with both active recharge and passive recharge for all included patients to determine side effect stimulation thresholds. Sixteen patients with 64 total DBS contacts met inclusion criteria for further analysis. Intraoperative monopolar review results were compared with the monopolar review from the first DBS programming visit.

**Results:**

The mean intraoperative active recharge stimulation threshold was 4.1 mA, while the mean intraoperative passive recharge stimulation threshold was 3.9 mA, though this difference was not statistically significant on *t*-test (*p* = 0.442). Mean stimulation threshold at clinic follow-up was 3.2 mA. In Pearson correlation, intraoperative passive recharge thresholds had stronger correlation with follow-up stimulation thresholds (Pearson *r* = 0.5281, *p* < 0.001) than intraoperative active recharge (Pearson *r* = 0.340, *p* = 0.018), however the difference between these correlations was not statistically significant on Fisher *Z* correlation test (*p* = 0.294). The mean difference between intraoperative passive recharge stimulation threshold and follow-up stimulation threshold was 0.8 mA, while the mean difference between intraoperative active recharge threshold and follow-up threshold was 1.2 mA. This difference was not statistically significant on a *t*-test (*p* = 0.134).

**Conclusions:**

Both intraoperative active recharge and passive recharge stimulation were well-correlated with the monopolar review at the first programming visit. No statistically significant differences were observed suggesting that either passive or active recharge may be utilized intraoperatively.

## 1 Introduction

Deep Brain Stimulation (DBS) is a well-established surgical therapy used to treat medication refractory disorders such as Parkinson's disease (PD), essential tremor (ET), and dystonia. While DBS can deliver promising clinical benefits, its therapeutic window can be reduced due to stimulation induced side effects. Consequently, patients may experience suboptimal DBS responses as well as persistent stimulation induced side effects ranging across motor and cognitive domains (Okun, 2000; Parsons et al., 2006; Follett et al., 2010).

**Figure 1:**
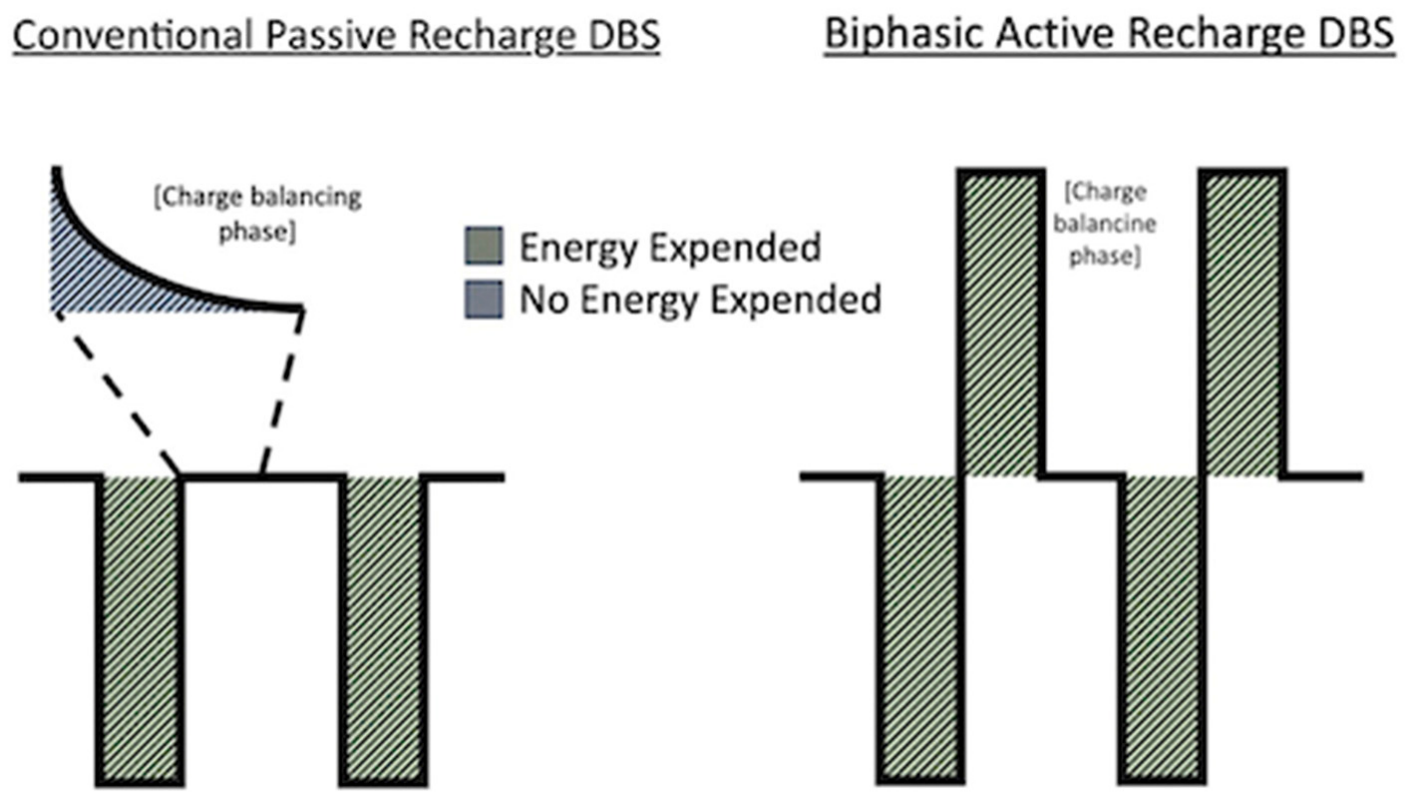
Conventional passive recharge DBS vs. biphasic active recharge DBS.

To further optimize patient outcomes after DBS and minimize its side effects, a variety of stimulation delivery strategies such as novel stimulation waveform shapes (Foutz and McIntyre, 2010; Hofmann et al., 2011), different pulse-width (Daniel et al., 2019; Dayal et al., 2020), frequency (Khoo et al., 2014; Su et al., 2018), and charge balancing systems have been used (De Jesus et al., 2018; Boogers et al., 2022). In particular, charge balancing is crucial to prevent net charge accumulation in the tissues and subsequent neural damage (Lilly et al., 1955), especially when delivering high-frequency stimulation as DBS (Piallat et al., 2009). Currently, DBS therapy from the implantable pulse generator (IPG) is delivered as a monophasic, square wave that relies on passive charge dissipation to neutral prior to the next pulse. This is known as passive recharge stimulation. An alternative charge balancing mechanism, active recharge, uses an active charge redistribution which requires additional battery power to deliver symmetric anodic and cathodic stimulation pulses to balance the net charge at the electrode-tissue interface (Figure 1). Compared to the monophasic passive recharge scheme of conventional DBS, biphasic active recharge can have differential clinical benefits by interfering in the depolarization-repolarization cycle of neurons (Almeida et al., 2017).

Several human studies have assessed the feasibility and clinical efficacy of active recharge, biphasic DBS in PD (Akbar et al., 2016; Okun, 2000; De Jesus et al., 2019), ET (Akbar et al., 2016; De Jesus et al., 2018; Boogers et al., 2022), and dystonia (Almeida et al., 2017; Wong et al., 2023). Compared to conventional passive recharge DBS systems, it has been shown that biphasic stimulation can deliver comparable clinical improvement. In some patients, biphasic stimulation may deliver similar or greater motor symptom improvement than traditional DBS therapy (Okun, 2000; De Jesus et al., 2019).

While passive recharge is the conventional stimulation paradigm in IPGs, intraoperative microelectrode recording platforms for awake DBS can deliver stimulation under a variety of paradigms. In this study, we sought to characterize differences between intraoperative active vs. passive recharge during the monopolar review with respect to stimulation induced side effects. Understanding the full extent of the therapeutic window of different stimulation paradigms is crucial to guide intraoperative decision making. Additionally, understanding the relationship between passive and active recharge can help optimize our intraoperative electrophysiologic testing to best represent anticipated therapeutic effects in the outpatient setting. In this retrospective study, we compared the side-effect stimulation thresholds between active and passive recharge strategies during DBS surgery through monopolar review and compared it to outpatient side effect thresholds.

## 2 Methods

The University of Florida Institutional Review Board (IRB) approved this study (IRB201901807).

### 2.1 Patient selection

Retrospective chart review was performed for 65 consecutive patients undergoing DBS lead implantation with a single attending neurologist (JW) from July 2021 through September 2022. Patient demographics, diagnosis, DBS target, and side effect stimulation thresholds during intraoperative monopolar review and postoperative monopolar review were collected. Intraoperative monopolar review was performed with both active recharge and passive recharge for all patients across each electrode contact.

### 2.2 Surgical procedure

Details of the surgical procedure have been previously published (Morishita et al., 2010). Briefly, a Cosman-Roberts-Wells (CRW) stereotactic frame was applied under local anesthesia and a stereotactic head CT scan was then obtained and fused to a pre-operative MRI. Targeting was carried out using our UF modified Schaltenbrand-Bailey atlas (Sudhyadhom et al., 2012). During the surgical procedure, microelectrode recording was used to define the target region. Medtronic DBS leads (Sensight 33005 or 33015 depending on the target) were implanted and macrostimulation testing was performed to evaluate stimulation-induced side effects. The monopolar review was conducted by systematically increasing stimulation amplitudes in 0.5 mA increments at each individual contact until a persistent side effect was noted (e.g., capsular side effect or sustained sensory side effect). The sensight leads were tested at each level in ring mode. During monopolar review patients were observed after symptom onset to determine whether a symptom was transient. Side effects were considered persistent if they did not abate within at least 10 s after onset and were reproducible with single-blind testing of amplitude changes. The threshold was then refined by 0.1 mA after initial side effect as observed. Pulse width and frequency were held at a constant 90 μs and 130 Hz for all patients. The maximum amplitude used during monopolar review was 6 mA. Monopolar review was performed using both active recharge and passive recharge settings on the Neuro Omega platform (Alpha Omega Engineering, Nof HaGalil, Israel), though active recharge was performed first as it is our conventional intraoperative testing protocol. The IPG was implanted ~4 weeks after lead implantation and activated during the first clinical DBS programming visit 1 week later. During this visit, conventional monopolar review was performed in clinic in the medication OFF state.

### 2.3 Statistical analysis

Threshold differences were calculated as [intraoperative active recharge threshold amplitude] – [intraoperative passive recharge threshold amplitude], as well as [intraoperative threshold amplitude] – [follow-up threshold amplitude]. A *t*-test was performed to compare mean intraoperative active and passive recharge thresholds. Pearson correlation was performed to correlate active recharge and passive recharge intraoperative thresholds with follow-up thresholds. Fisher *Z* test correlation test was performed to compare the two Pearson correlations. A Bonferroni adjusted *p*-value of 0.0125 was used to correct for multiple comparisons.

## 3 Results

**Figure 2:**
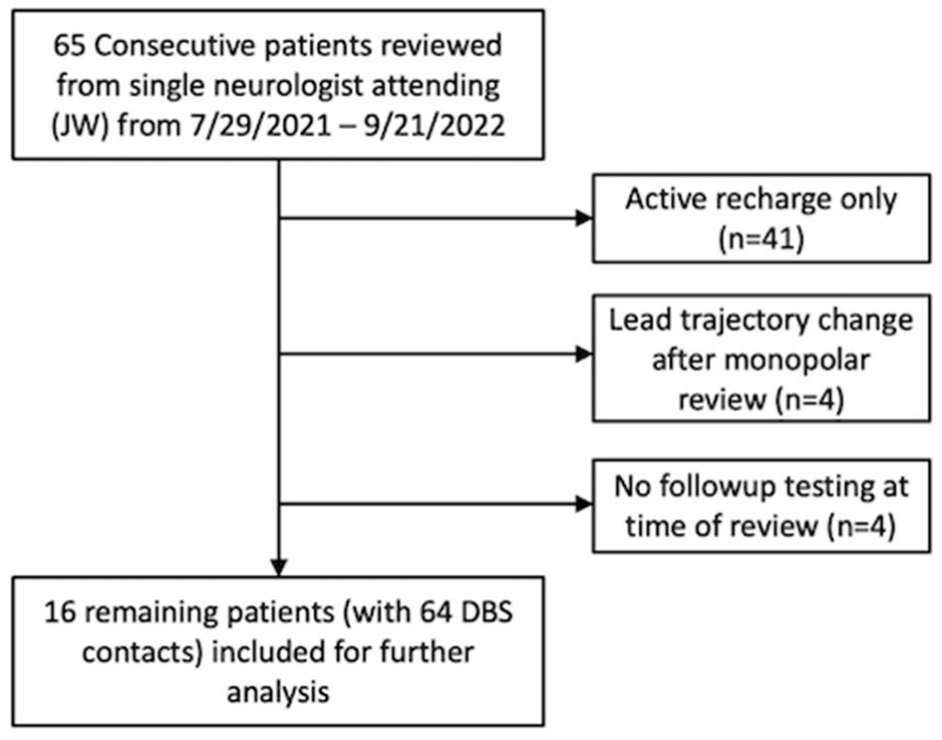
Flowsheet of patients excluded and included for analysis.

There were 65 consecutive patients who underwent DBS with a single attending neurologist (JW) from July 2021 through September 2022. Patients were excluded if only active recharge was performed (*N* = 41), lead trajectory was changed after monopolar review (*n* = 4), or if there was no follow-up testing at time of review (*n* = 4). The resulting 16 patients with 64 total DBS contacts were included for further analysis (Figure 2). Contacts where no side effects were experienced at maximum stimulation amplitude were excluded for analysis.

### 3.1 Patient characteristics

Patient characteristics are displayed in Table 1. The mean age was 66 years old, and five (31%) patients were female. The most common condition treated was PD (12 patients, 75%), and the most common target was the globus pallidus interna (GPi; 10 patients, 62.5%). Mean intraoperative side effect thresholds obtained by active recharge were 4.1 mA (± 1.3), while mean intraoperative thresholds obtained by passive recharge were 3.9 mA (± 1.3). Mean thresholds at first follow-up visit were 3.2 mA (± 1.2).

**Table 1:** Patient characteristics for the 16 included patients (64 DBS contacts).

***N* = 16 patients, 64 DBS contacts**	**Mean (±SD), *N* (%)**
Age	66 (± 6.8)
**Pathology**	
PD	12 (75%)
ET	2 (12.5%)
Dystonia	2 (12.5%)
Female	5 (31%)
**Target**	
GPi	10 (62.5%)
STN	4 (25%)
VIM	2 (12.5%)
Side (left)	10 (62.5%)
Intraoperative active recharge threshold (*n* = 50 contacts)	4.1 mA (± 1.3)
Intraoperative passive recharge threshold (*n* = 54 contacts)	3.9 mA (± 1.3)
Follow-up thresholds (*n* = 59 contacts)	3.2 mA (± 1.2)

ET, essential tremor; PD, Parkinson's disease; GPi, Globus pallidus internus; STN, subthalamic nucleus; VIM, ventralis intermediate nucleus; SD, standard deviation.

Contacts where no side effects were experienced at maximum stimulation amplitude were excluded for analysis.

### 3.2 Active recharge vs. passive recharge thresholds

**Figure 3:**
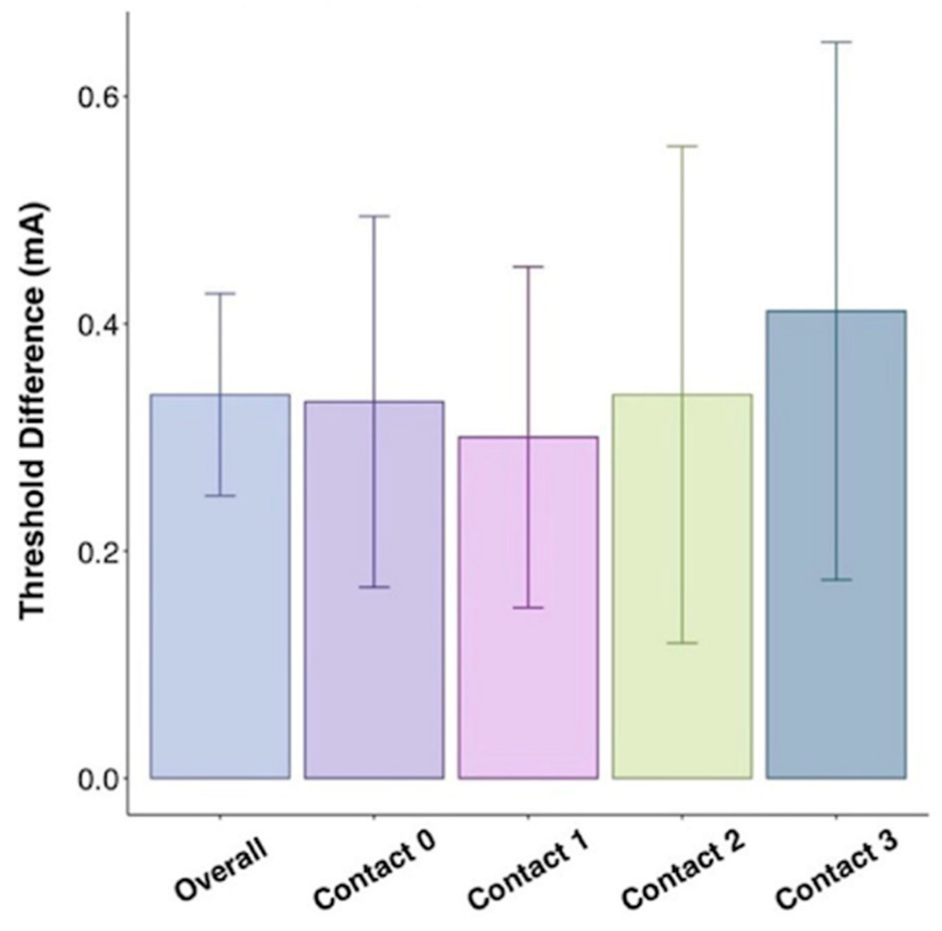
Threshold difference between intraoperative active recharge threshold and passive recharge threshold by contact with standard error bars.

While mean active recharge intraoperative thresholds tended to be higher than passive recharge (4.1 vs. 3.9 mA), this difference was not statistically significant on *t*-test (*p* = 0.442; Figure 3). This difference was also not significant when tested at Contact 0 (*p* = 0.296), Contact 1 (*p* = 0.312), Contact 2 (*p* = 0.878), or Contact 3 (*p* = 0.877).

### 3.3 Association with post-operative thresholds

**Figure 4:**
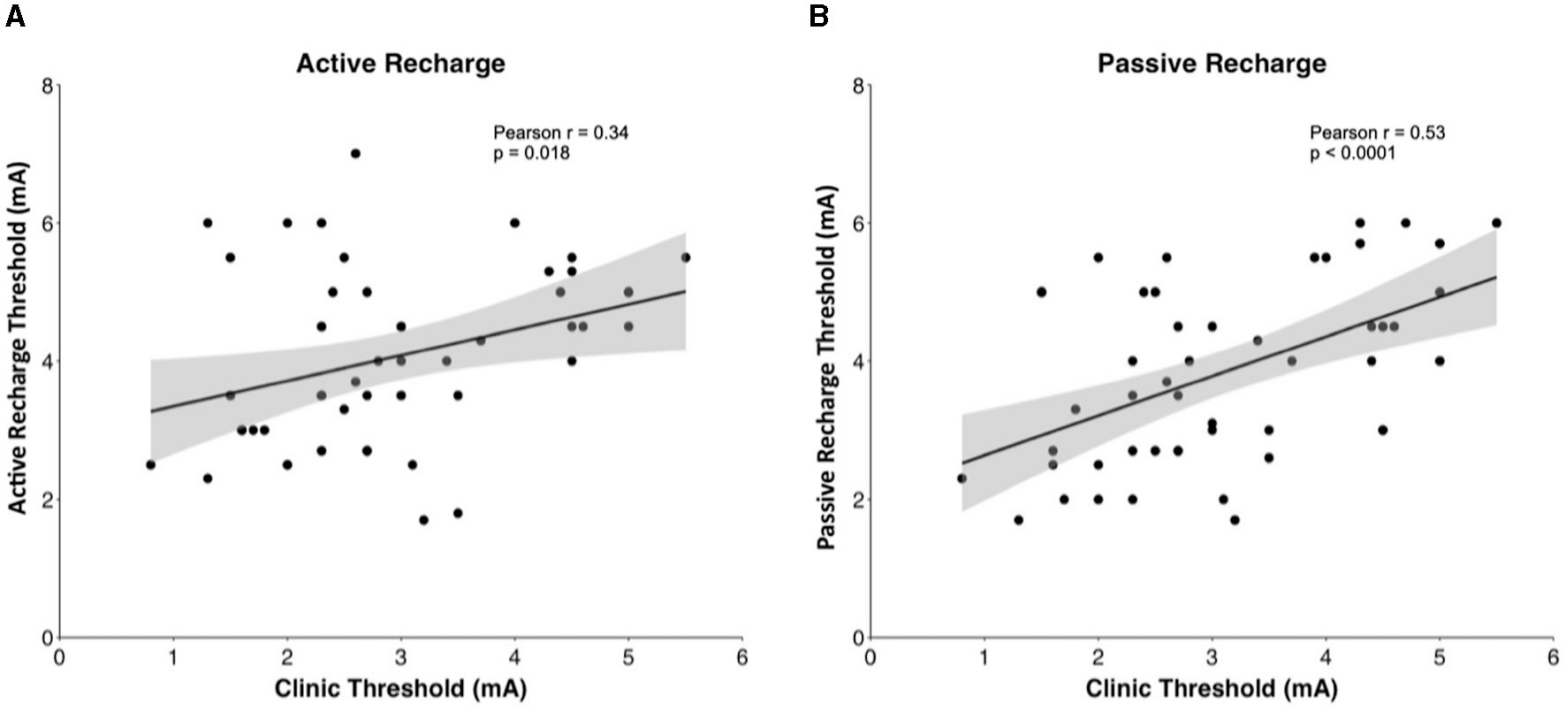
Pearson correlation between **(A)** intraoperative active recharge threshold and clinic thresholds and **(B)** intraoperative passive recharge threshold with 95% confidence interval in gray.

**Figure 5:**
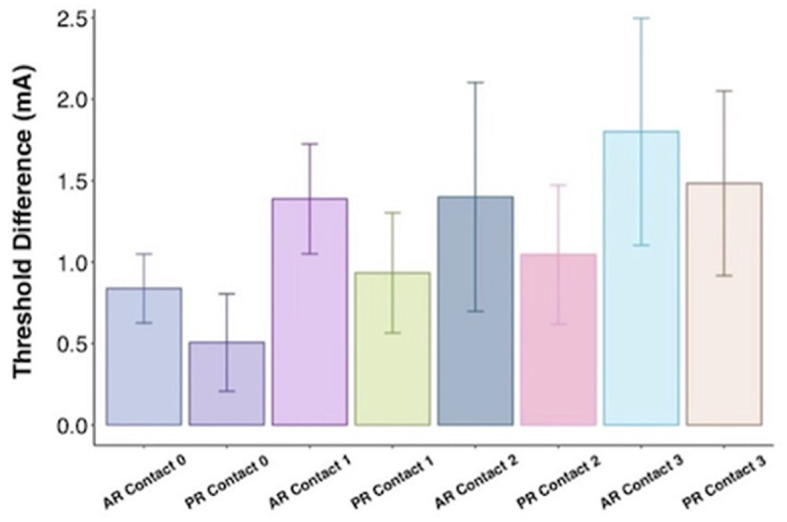
Threshold difference between intraoperative threshold and chronic threshold by charge balancing setting (AR, active recharge; PR, passive recharge) and contact with standard error bars.

In Pearson correlation, intraoperative passive recharge thresholds had a stronger correlation with chronic passive recharge stimulation thresholds (Pearson *r* = 0.5281, *p* < 0.001) than intraoperative active recharge stimulation thresholds (Pearson *r* = 0.340, *p* = 0.018; Figure 4), however the difference between these correlations was not statistically significant on Fisher *Z* test correlation test (*p* = 0.294). The mean difference between intraoperative passive recharge stimulation thresholds and chronic passive recharge stimulation thresholds was 0.8 mA, while the mean difference between intraoperative active recharge thresholds and chronic passive recharge thresholds was 1.2 mA, however this difference was not statistically significant on *t*-test (*p* = 0.134). Comparing the threshold difference between intraoperative and chronic thresholds with active vs. passive recharge settings was also not significant when comparing at Contact 0 (*p* = 0.373), Contact 1 (*p* = 0.370), Contact 2 (*p* = 0.654), or Contact 3 (*p* = 0.725; Figure 5).

## 4 Discussion

In this study, we found that side effect thresholds obtained by intraoperative passive recharge had a stronger correlation to chronic outpatient follow-up thresholds compared to intraoperative active recharge. While both stimulation paradigms correlated well with chronic postoperative side effect thresholds, the correlation was stronger for the passive recharge setting. When comparing the correlations for each intraoperative stimulation paradigm, the difference was not statistically significant, however our study was likely underpowered to find a statistical difference.

Our observations suggest that intraoperative passive recharge could potentially be used in place of the conventional Neuro Omega active recharge setting intraoperatively. By using intraoperative passive recharge, we may be able to better represent the anticipated clinical effects that patients experience in the postoperative setting. This knowledge on the reliability of our intraoperative patient assessments can help guide treatment decisions such as predictive value of procedure efficacy and informing potential trajectory changes. This is the first study to compare how different intraoperative stimulation paradigms correlate with chronic stimulation settings.

In this study, we also found that active recharge tended to produce side effect thresholds at greater amplitudes compared to passive recharge, as only seven of the 64 contacts had passive recharge thresholds that were higher than active recharge. This difference is likely due to the wider distribution of charge in the neural tissue. This difference was observed at each contact, however with a limited sample size, statistical significance was lacking. Additionally, intraoperative side effect thresholds tend to be higher than postoperative side effect thresholds, regardless of whether they are obtained by passive or active recharge. This is likely due to the effects of acute cerebral edema and inflammation at the electrode-tissue interface during lead placement which increases impedance, as well as the controlled outpatient setting which may allow for increased time and sensitivity of side effect testing (Wong et al., 2018).

Overall, active recharge produces higher side effect thresholds, suggesting that the utility of chronic active recharge paradigms (available on rechargeable implantable pulse generators) could provide an enhanced stimulation paradigm in patients with narrow therapeutic windows. Similarly, Boogers et al. recently evaluated side effect thresholds between different stimulation paradigms and found that biphasic stimulation pulses had a wider therapeutic window and higher side effect threshold compared to traditional stimulation, and that that these differences were most pronounced for anodic-first biphasic pulses (Boogers et al., 2022). Our findings are consistent with the findings from Boogers et al. Future studies with larger cohorts involving different stimulation patterns are needed to further assess optimal stimulation patterns and outcomes.

### 4.1 Limitations

Our study carries with it the inherent limitations of a retrospective review. While patients acted as their own controls in this study, receiving both intraoperative stimulation paradigms, our study is not powered to detect differences by underlying pathology or brain target. Different brain targets have previously shown differential responses to stimulation so future studies should investigate different stimulation paradigms by target (Wong et al., 2018). Additionally, this is a single center study which may limit the generalizability of our findings. Future studies may be aided by larger cohorts and prospective manner through which active and passive recharge threshold testing could be randomized and compared to clinic thresholds.

## 5 Conclusions

Both intraoperative active recharge and passive recharge stimulation were well-correlated with the monopolar review at the first programming visit. No statistically significant differences were observed suggesting that either passive or active recharge may be utilized intraoperatively.

## Data availability statement

The raw data supporting the conclusions of this article will be made available by the authors, without undue reservation.

## Ethics statement

The studies involving humans were approved by University of Florida Institutional Review Board. The studies were conducted in accordance with the local legislation and institutional requirements. The Ethics Committee/institutional review board waived the requirement of written informed consent for participation from the participants or the participants' legal guardians/next of kin because the study was a retrospective chart review without risk to participants.

## Author contributions

DM: Data curation, Formal analysis, Investigation, Methodology, Writing—original draft, Writing—review & editing. MK: Writing—original draft, Writing—review & editing. TO: Writing—original draft, Writing—review & editing. ZS: Investigation, Writing—original draft, Writing—review & editing. VC: Investigation, Writing—original draft, Writing—review & editing. CC: Investigation, Writing—original draft, Writing—review & editing. RZ-F: Investigation, Writing—original draft, Writing—review & editing. JH: Formal analysis, Investigation, Methodology, Software, Supervision, Writing—original draft, Writing—review & editing. JW: Conceptualization, Formal analysis, Investigation, Methodology, Software, Supervision, Writing—original draft, Writing—review & editing.
